# Supplementary material for: Multimodal imaging and functional analysis of the chick NMDA retinal damage model
Source: PLoS One. 2021 Sep 7;16(9):e0257148. doi: 10.1371/journal.pone.0257148 (PMC8423281; doi:10.1371/journal.pone.0257148)
Supplement: S1 File — (ZIP) [file pone.0257148.s004.zip › SD-OCT/C200727/010/D1-PWk1/C200727-010_OD_R_14_0_REGAVG0000016/Manual_PreDefineThreeByFourGridRadialRetina_1.pdf]

Three By Four Grid Radial Retina Template Report

Patient ID	C200727	Name	See InVivoVue
Exam Date	See InVivoVue	Exam Time	See InVivoVue
Eye	OD	Averaged	N/A
Depth (mm)	1.65	Depth Samples	1024
Pitch (um/pix)	14.000 x 2333.333 x 1.608	Refractive Index	See InVivoVue
Scan Type	Radial 14.000mm x 14.000mm x 1.647mm , 1000 x 6 x 1024		

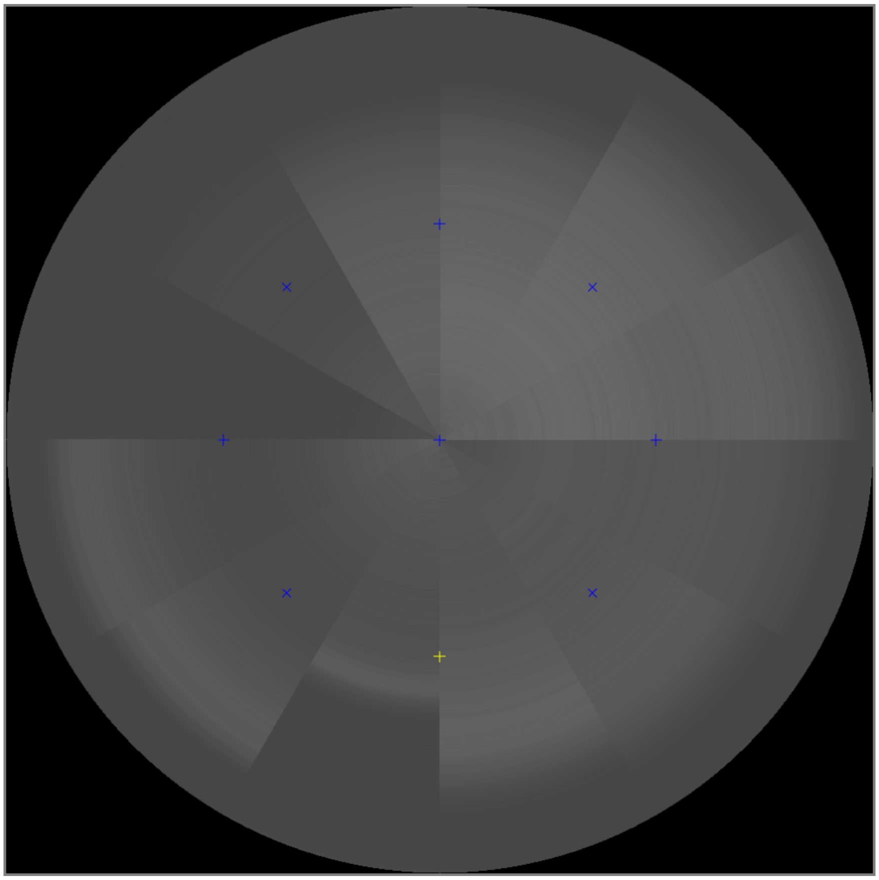

Thickness Metrics

Analysis	Average(mm)	Minimum(mm)	Maximum(mm)	STDEV(mm)
IRNFL - RPE	0.2855	0.2758	0.3033	0.0100
IRNFL - ORNFL	0.0465	0.0212	0.0917	0.0244
ORNFL - OPL	0.1660	0.1354	0.1910	0.0201
OPL - ETPRS	0.0559	0.0530	0.0599	0.0029
